# Supplementary figures and images for: Triclosan depletes the membrane potential in Pseudomonas aeruginosa biofilms inhibiting aminoglycoside induced adaptive resistance
Source: PLoS Pathog. 2020 Oct 30;16(10):e1008529. doi: 10.1371/journal.ppat.1008529 (PMC7657502; doi:10.1371/journal.ppat.1008529)

## Slide 1
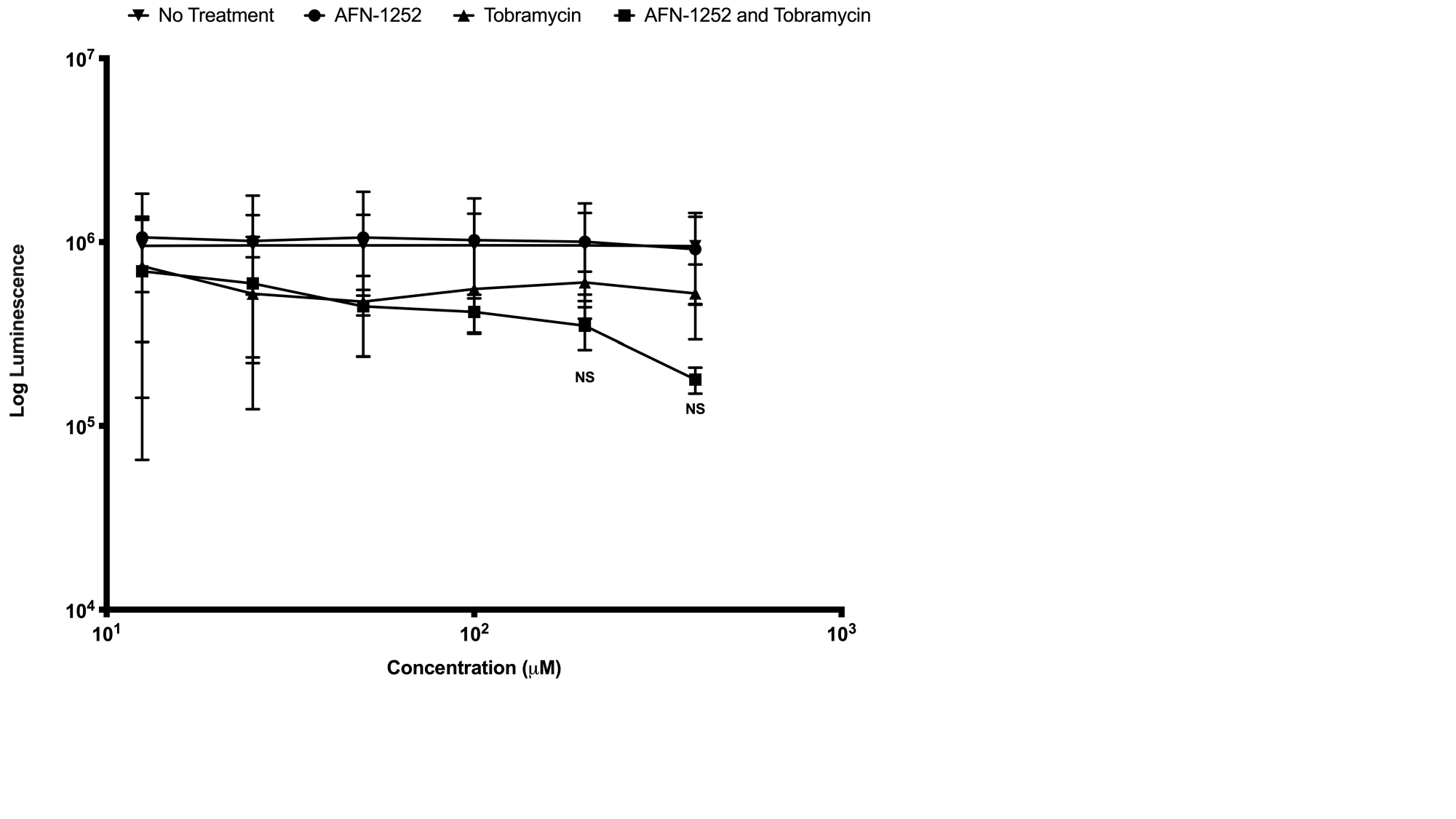

Supplement: S1 Fig — 24-hr old biofilms grown on MBEC plates were treated for 6-hrs with AFN-1252 (400 μM), tobramycin (400 μM), alone and in combination in two-fold dilutions, and the number of viable cells within the biofilms were quantified by BacTiter-GloTM. The assay was performed twice in in duplicate. The results represent means ± the standard deviation (SD). A one-way analysis of variance (ANOVA) followed by Bonferroni’s multiple comparison post-hoc test was used to determine statistical significance between tobramycin versus the combination (NS, not significant). (PPTX) [file ppat.1008529.s001.pptx]

## Slide 1
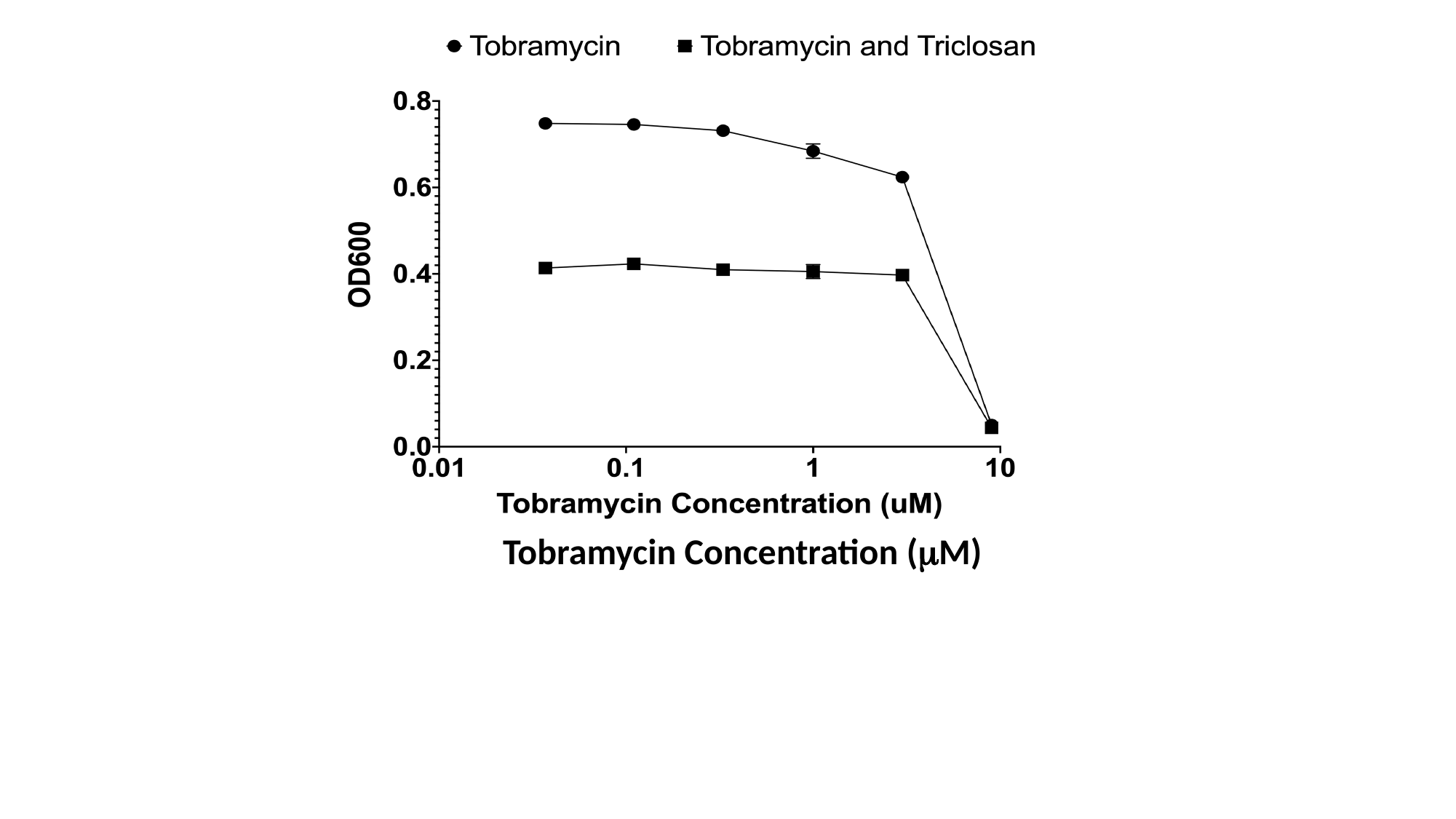

Tobramycin Concentration (mM)

Supplement: S2 Fig — Planktonic cells were treated with the indicated concentrations of tobramycin with or without 100 μM triclosan and grown for 16 hours before measuring the OD600. Error bars indicate the standard deviation. (PPTX) [file ppat.1008529.s002.pptx]

## Slide 1
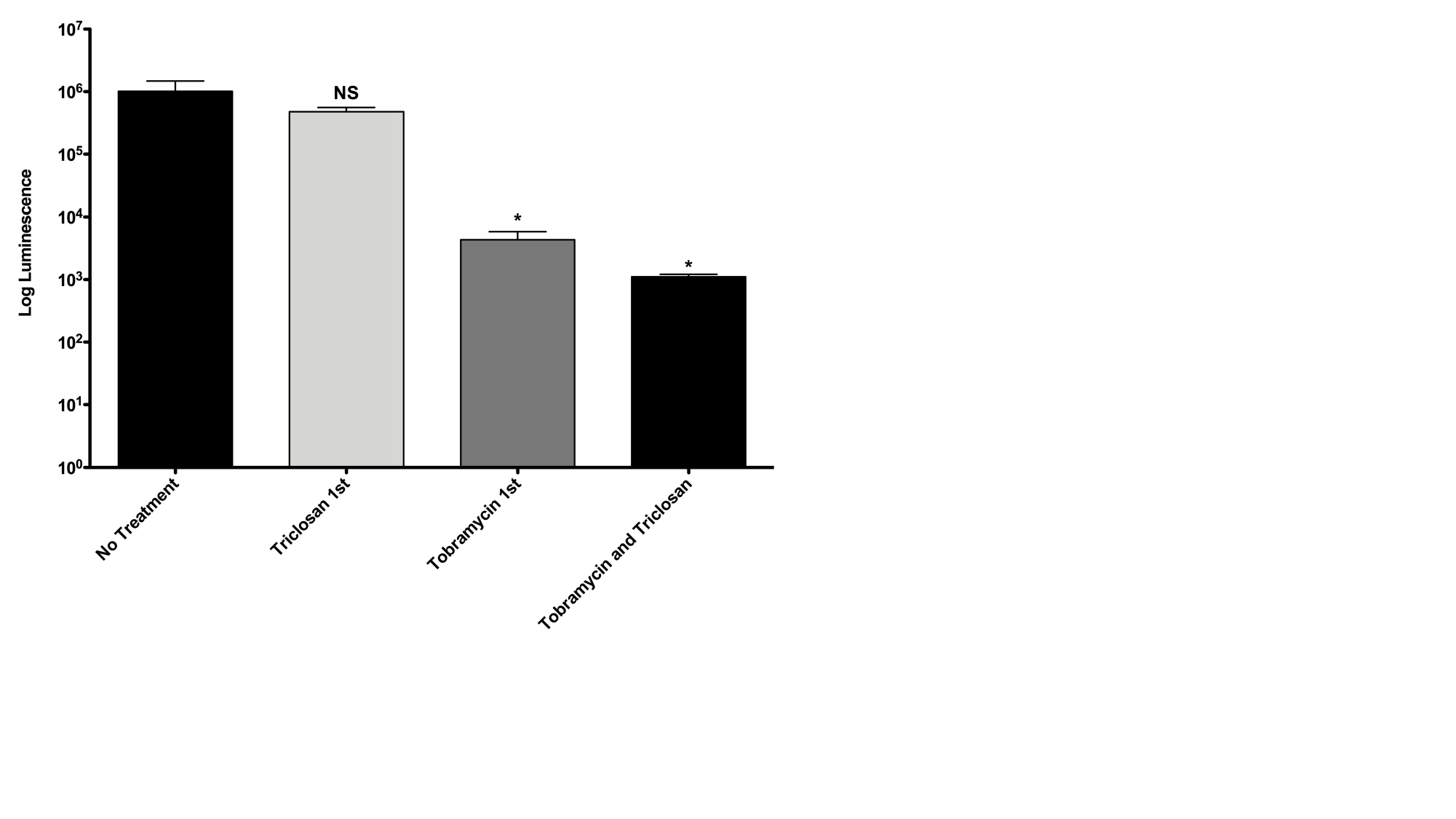

Supplement: S3 Fig — 24-hr old biofilms grown on MBEC plates were treated sequentially. First, biofilms were treated for 3-hours with tobramycin (500 μM) and then washed three times in DPBS for 3-mins each, before being treated with triclosan (100 μM) for 3-hours or vice versa. As a control, biofilms were also treated for 6-hrs with triclosan and tobramycin. The number of viable cells within the biofilms were quantified by BacTiter-GloTM. The assay was performed twice in in duplicate. The results represent means plus the SD. A one-way ANOVA followed by Tukey’s multiple comparison post-hoc test was used to determine statistical significance between each treatment and the untreated control. *, p<0.05. NS, not significant. (PPTX) [file ppat.1008529.s003.pptx]

## Slide 1
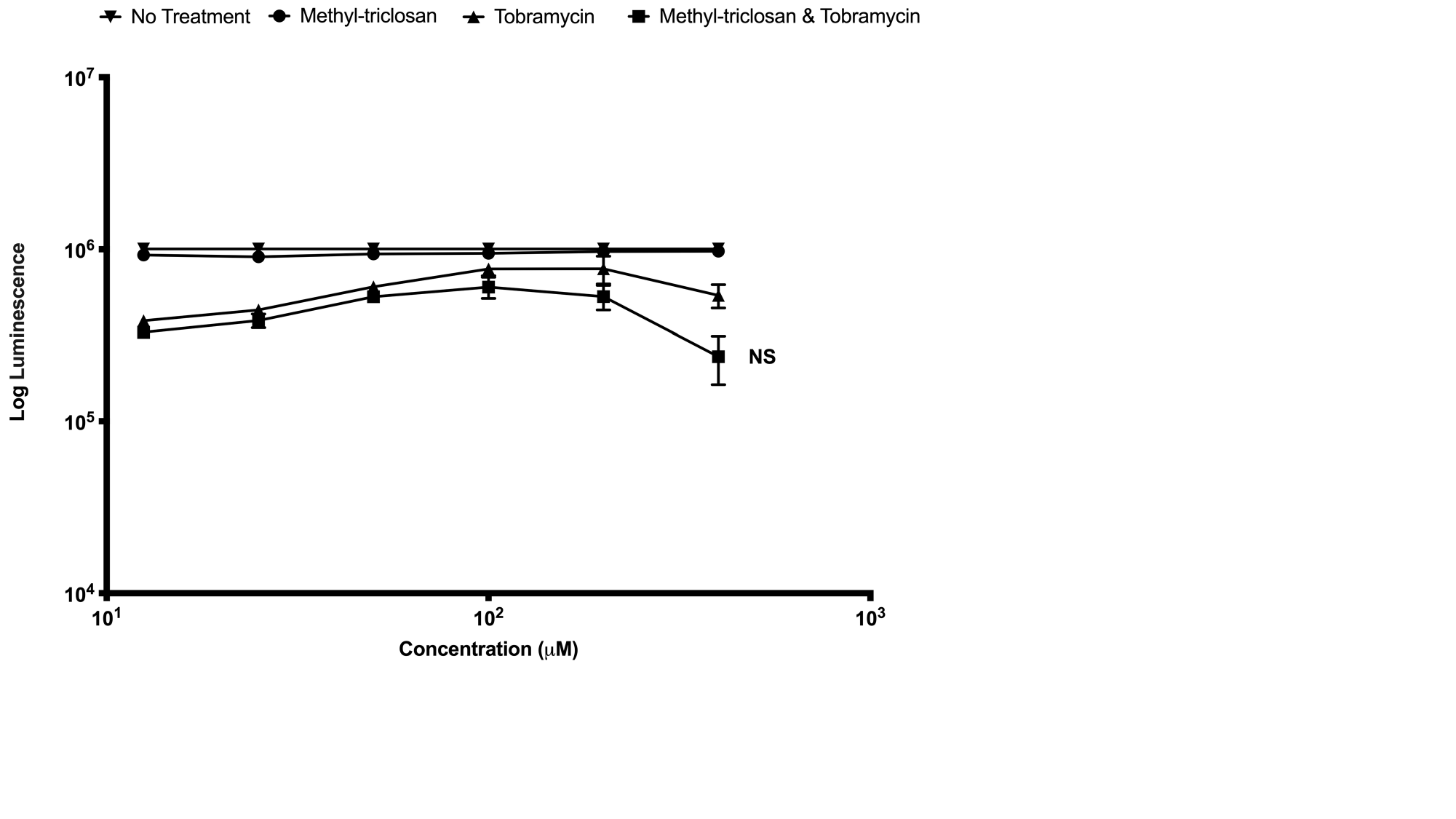

Supplement: S5 Fig — 24-hr old biofilms grown on MBEC plates were treated for 6-hrs with methyl-triclosan (100 μM), tobramycin (400 μM), alone and in combination in two-fold dilutions, and the number of viable cells within the biofilms were quantified by BacTiter-GloTM. The assay was performed twice in in duplicate. The results represent means ±SD. A one-way ANOVA followed by Bonferroni’s multiple comparison post-hoc test was used to determine statistical significance compared to tobramycin alone and the combination. NS, not significant. (PPTX) [file ppat.1008529.s005.pptx]

## Slide 1
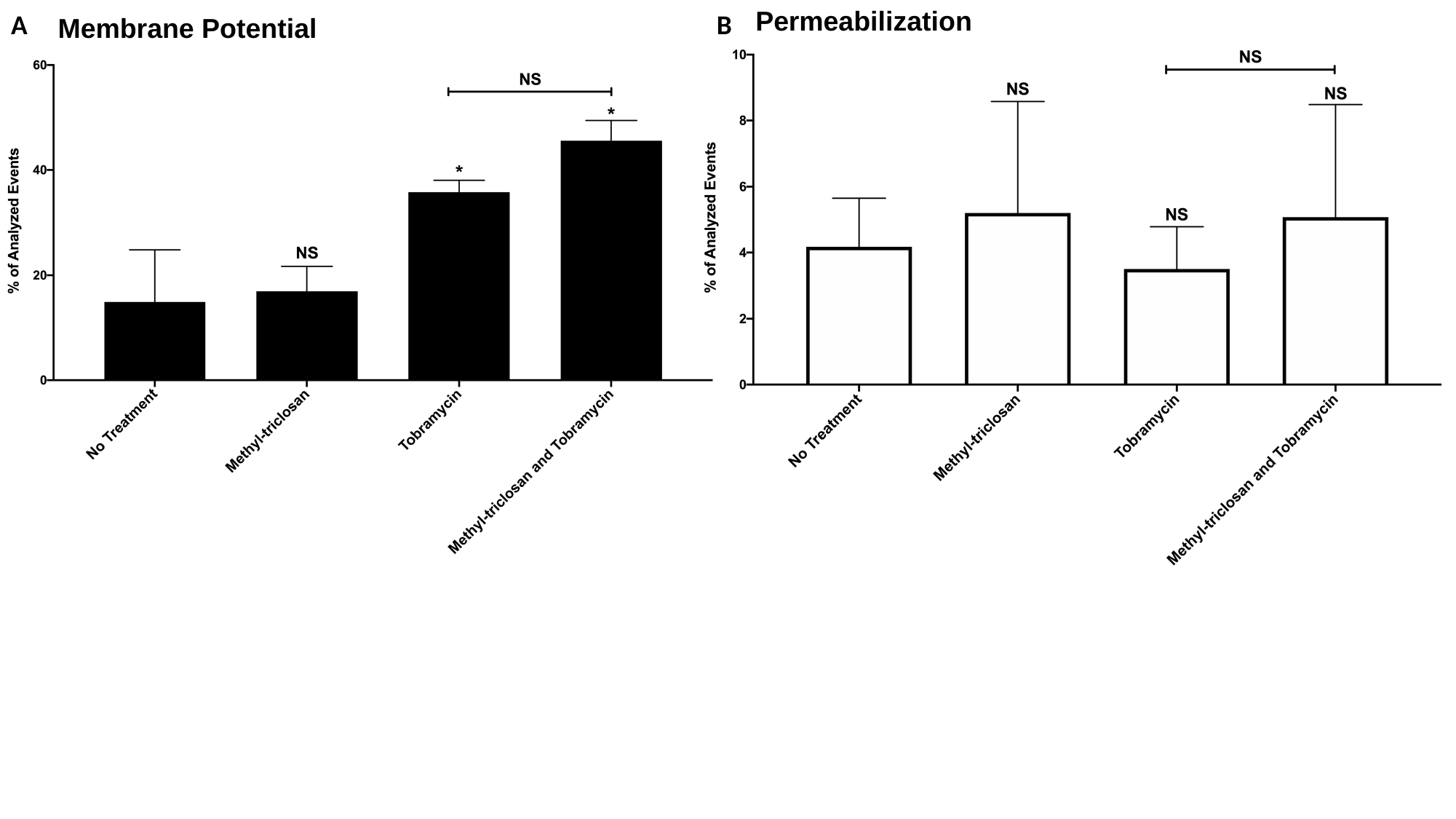

Permeabilization
B
A
Membrane Potential

Supplement: S6 Fig — 24-hr old biofilms were treated with methyl-triclosan (100 μM), or tobramycin (500 μM), alone and in combination for 2-hrs. Cells were stained with DiOC2(3) and TO-PRO™-3 iodide to determine the number of cells that maintained a membrane potential (A) or were permeabilized (B), respectively. Dead or permeabilized cells were excluded from membrane potential analysis and are shown in panel B. The experiment was performed two separate times in duplicate. The results are percent averages plus the SD. Percent values indicate the average relative abundance of events within each gate normalized to the total number of events analyzed, excluding artifacts, aggregates and debris. A one-way ANOVA followed by Dunnett’s multiple comparison post-hoc test was used to determine statistical significance between each group and the untreated control and Bonferroni’s post-hoc test was performed to compare tobramycin vs tobramycin and methyl-triclosan. *, p<0.05. NS, not significant. (PPTX) [file ppat.1008529.s006.pptx]

## Slide 1
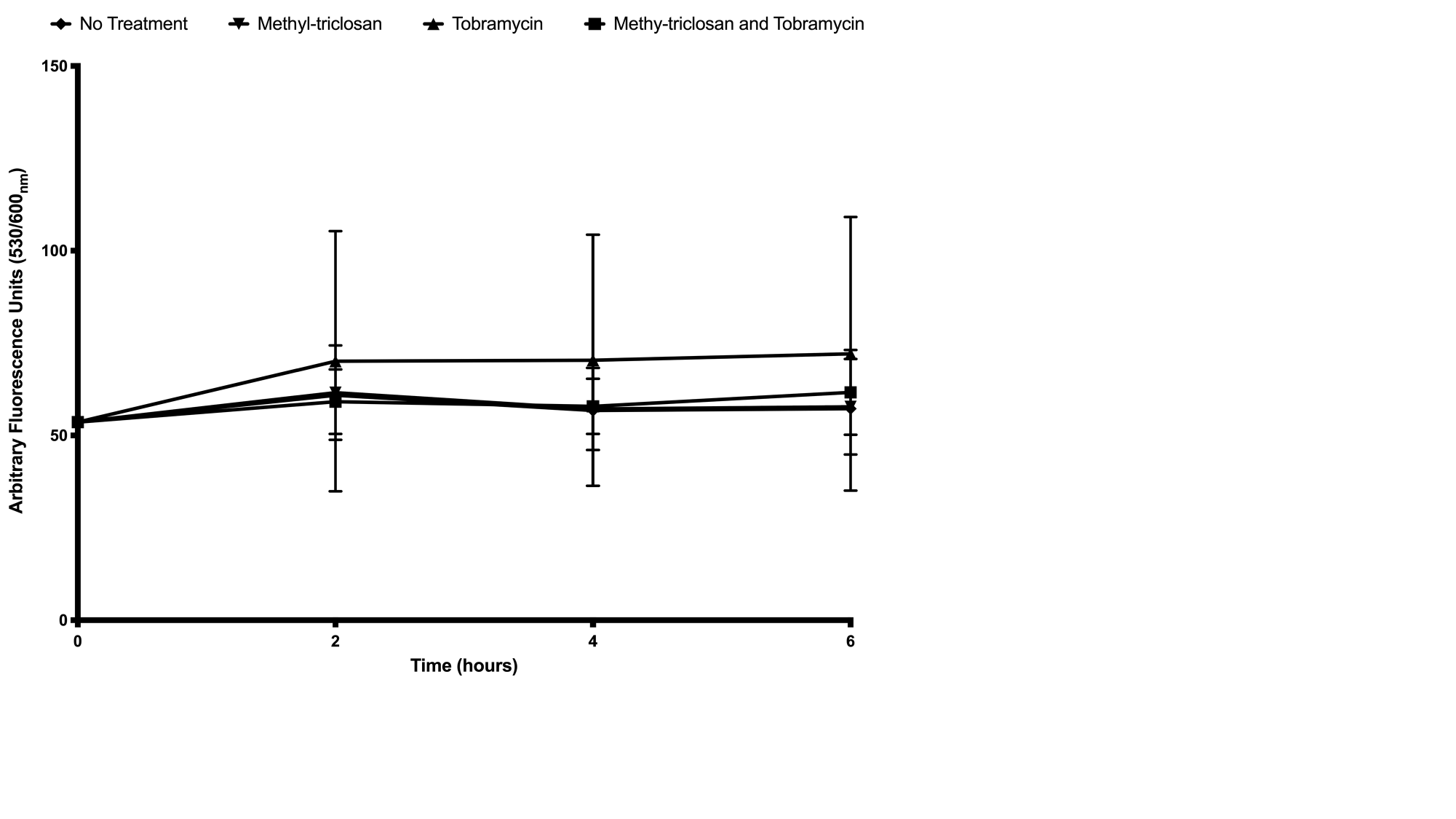

Supplement: S7 Fig — Ethidium bromide is a substrate of RND-type efflux pumps. 24-hr biofilms were stained with ethidium bromide to measure accumulation. Biofilms were treated with methyl-triclosan (100 μM) and tobramycin (500 μM) alone and in combination. Fluorescence was read at 0,2,4, and 6-hrs. The assay was performed three times in triplicate. Results represent the average arbitrary fluorescence units ±SD. (PPTX) [file ppat.1008529.s007.pptx]

## Slide 1
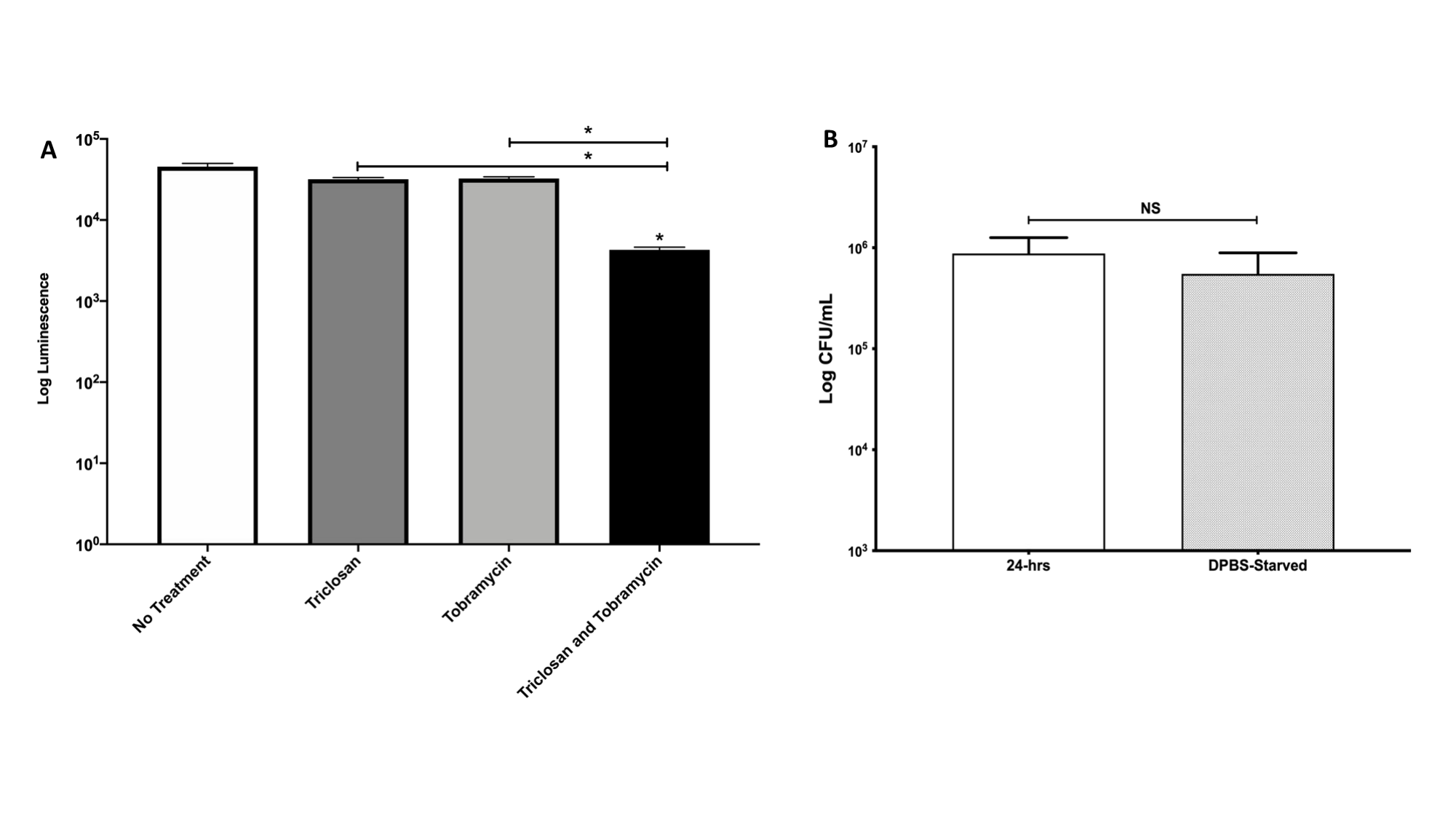

Supplement: S8 Fig — 24-hour old biofilms were starved of nutrients by replacing media with DPBS for 5-days. Starved biofilms were treated with triclosan (100 μM), or tobramycin (500 μM), alone and in combination for 6-hrs. The number of viable cells within the biofilms was quantified using the BacTiter-Glo™ assay. The assay was performed once using three biological replicates. The results represent the means plus the SEM. A one-way ANOVA followed by Sidak’s multiple comparison post hoc test was used to determine statistical significance between the combination treatment and the untreated control and between triclosan alone or tobramycin alone and the combination treatment as indicated by the bars. *, P < 0.05. Colony forming units (CFUs)/mL were calculated for 24-hr biofilms and biofilms that had been starved in DPBS for 5-days. A t-test was performed to determine the statistical difference between mature biofilms and starved biofilms, NS, not significant. (PPTX) [file ppat.1008529.s008.pptx]

## Slide 1
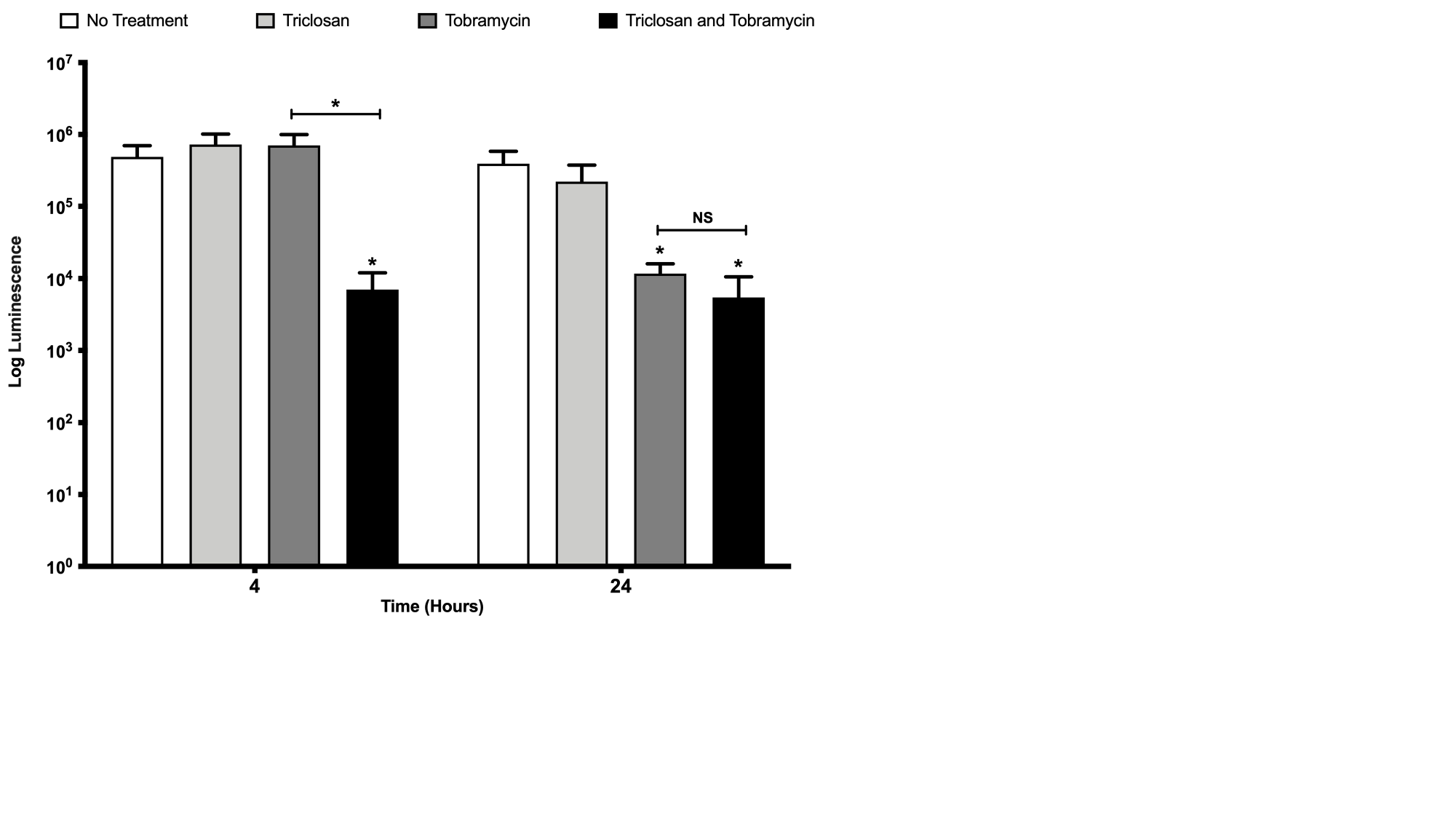

Supplement: S9 Fig — 24-hr old biofilms were treated for 24-hrs with triclosan (100 μM), tobramycin (500 μM), alone and in combination, and the number of viable cells within the biofilms were quantified by BacTiter-GloTM. The assay was performed at least three times in triplicate. The results represent means plus the SEM. A two-way ANOVA followed by Tukey’s multiple comparison post-hoc test was used to determine statistical significance between each group. *, P < 0.05. NS, not significant. 4-hr treatments were previously published and are shown for comparison [28]. (PPTX) [file ppat.1008529.s009.pptx]
