# Supplementary material for: Triclosan depletes the membrane potential in Pseudomonas aeruginosa biofilms inhibiting aminoglycoside induced adaptive resistance
Source: PLoS Pathog. 2020 Oct 30;16(10):e1008529. doi: 10.1371/journal.ppat.1008529 (PMC7657502; doi:10.1371/journal.ppat.1008529)
Supplement: S4 Fig — Untreated 24-hr old biofilms were stained with TO-PRO™-3 to determine the number of cells that were permeabilized. Cells were also stained with DiOC2(3) to determine the number of cells maintaining a membrane potential. A. Scatter plot of side scatter area (SSC-A) versus forward scatter area (FSC-A). Gate P1 excludes debris and artifacts. B. Histogram plot of count versus APC from P1 gate shown. Gate P2 indicates cells that are TO-PRO™-3 negative because they have an intact outer membrane (OM), preventing the diffusion of TO-PRO™-3 into the cell. Gate P3 indicates cells that stain positive for TO-PRO™-3, indicating permeabilization, thus allowing TO-PRO™-3 to diffuse into the cytosol and bind DNA emitting in the APC channel. C. Cells with intact OMs from population P2 were plotted as a scatter plot of PE-A versus FITC-A. Gate P4 indicates cells that stain positive with DiOC2(3), which emits in the FITC channel in all cells. Gate P5 indicates cells with higher membrane potentials, which drives the accumulation of DiOC2(3) within the cytosol, subsequently resulting in self-association and a shift in fluoresces to the PE channel. (PPTX) [file ppat.1008529.s004.pptx]

## Slide 1
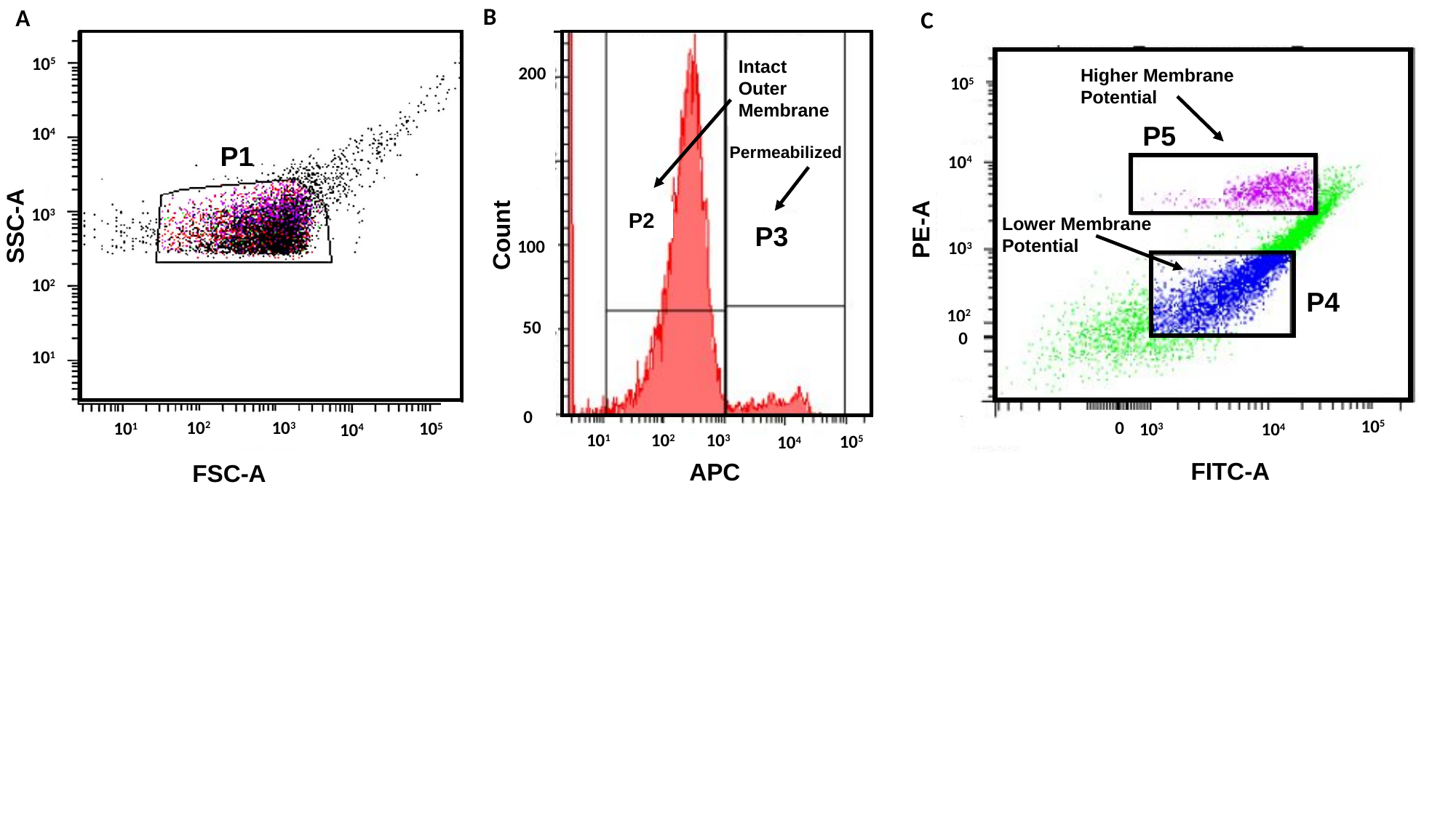

B
A
C
105
Intact
Outer
Membrane
200
Higher Membrane Potential
105
P5
104
P1
Permeabilized
104
PE-A
SSC-A
Count
103
P2
Lower Membrane Potential
P3
100
103
102
P4
102
50
0
101
0
105
102
0
103
105
101
103
104
104
102
101
103
105
104
FITC-A
APC
FSC-A
