# Supplementary material for: Triclosan depletes the membrane potential in Pseudomonas aeruginosa biofilms inhibiting aminoglycoside induced adaptive resistance
Source: PLoS Pathog. 2020 Oct 30;16(10):e1008529. doi: 10.1371/journal.ppat.1008529 (PMC7657502; doi:10.1371/journal.ppat.1008529)
Supplement: S1 Table — Gradual and moderate treatment groups were started at different concentrations of tobramycin and triclosan. However, they were then treated in subsequent cycles with ever increasing concentrations of triclosan and tobramycin at the same rate. For example, the moderate treatment group reached 1 μM of tobramycin and 100 μM of triclosan in 8-cycles, whereas the gradual treatment group reached the same concentration in 16-cycles from their initial treatments. The sudden treatment series was treated with 500 μM of triclosan and tobramycin from cycle day 1 and was eradicated, thus, is not shown in the table. Highlighted in yellow, the minimum inhibitory concentration (MIC) of tobramycin against planktonic cells was found to be 1μM and is shown. (PPTX) [file ppat.1008529.s010.pptx]

## Slide 1
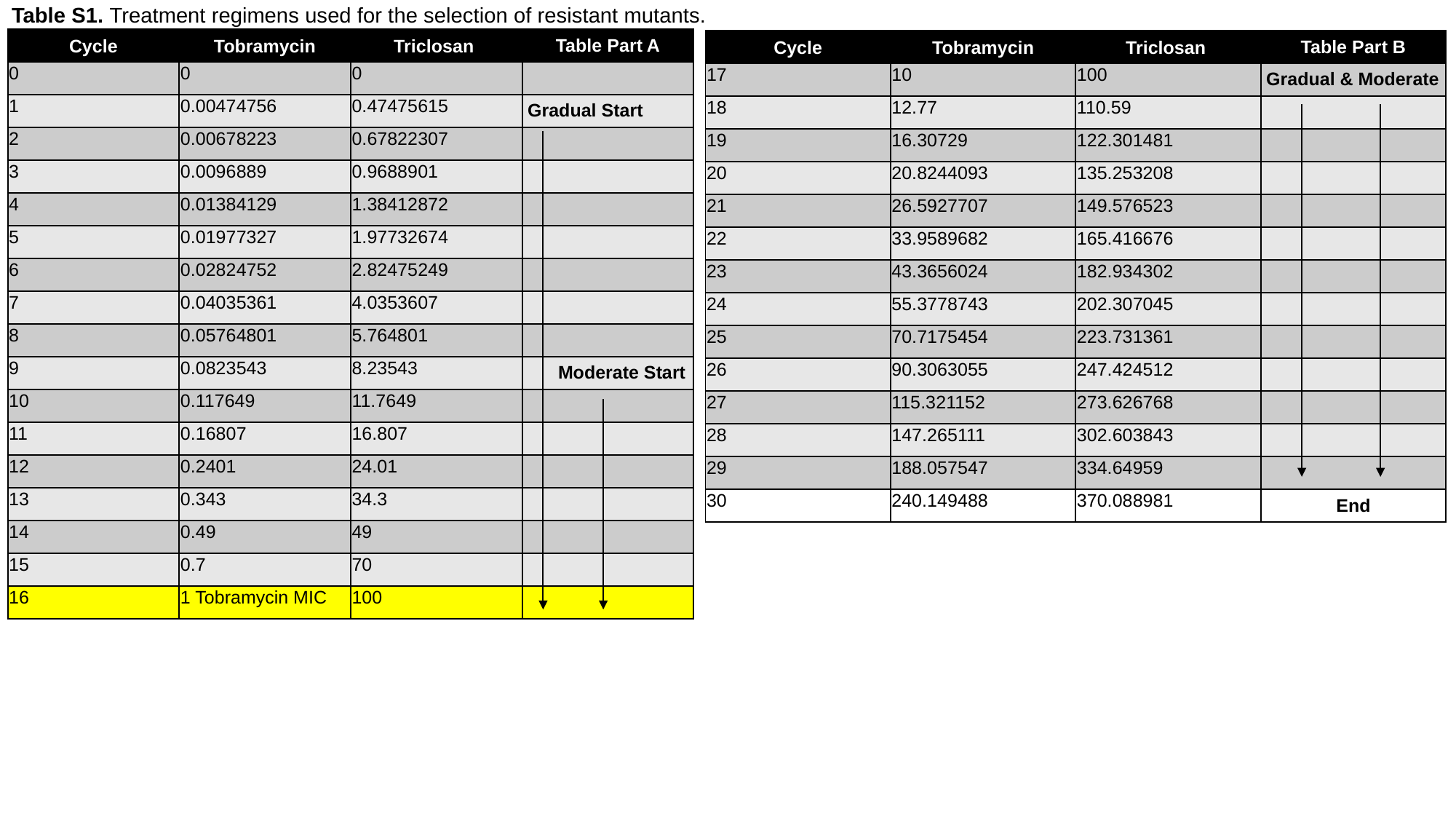

Table S1. Treatment regimens used for the selection of resistant mutants.
| Cycle | Tobramycin | Triclosan | Table Part A |
| --- | --- | --- | --- |
| 0 | 0 | 0 | |
| 1 | 0.00474756 | 0.47475615 | Gradual Start |
| 2 | 0.00678223 | 0.67822307 | |
| 3 | 0.0096889 | 0.9688901 | |
| 4 | 0.01384129 | 1.38412872 | |
| 5 | 0.01977327 | 1.97732674 | |
| 6 | 0.02824752 | 2.82475249 | |
| 7 | 0.04035361 | 4.0353607 | |
| 8 | 0.05764801 | 5.764801 | |
| 9 | 0.0823543 | 8.23543 | Moderate Start |
| 10 | 0.117649 | 11.7649 | |
| 11 | 0.16807 | 16.807 | |
| 12 | 0.2401 | 24.01 | |
| 13 | 0.343 | 34.3 | |
| 14 | 0.49 | 49 | |
| 15 | 0.7 | 70 | |
| 16 | 1 Tobramycin MIC | 100 | |
| Cycle | Tobramycin | Triclosan | Table Part B |
| --- | --- | --- | --- |
| 17 | 10 | 100 | Gradual & Moderate |
| 18 | 12.77 | 110.59 | |
| 19 | 16.30729 | 122.301481 | |
| 20 | 20.8244093 | 135.253208 | |
| 21 | 26.5927707 | 149.576523 | |
| 22 | 33.9589682 | 165.416676 | |
| 23 | 43.3656024 | 182.934302 | |
| 24 | 55.3778743 | 202.307045 | |
| 25 | 70.7175454 | 223.731361 | |
| 26 | 90.3063055 | 247.424512 | |
| 27 | 115.321152 | 273.626768 | |
| 28 | 147.265111 | 302.603843 | |
| 29 | 188.057547 | 334.64959 | |
| 30 | 240.149488 | 370.088981 | End |
