# Supplementary material for: Triclosan depletes the membrane potential in Pseudomonas aeruginosa biofilms inhibiting aminoglycoside induced adaptive resistance
Source: PLoS Pathog. 2020 Oct 30;16(10):e1008529. doi: 10.1371/journal.ppat.1008529 (PMC7657502; doi:10.1371/journal.ppat.1008529)
Supplement: Attachment — Submitted filename: Plos_Response-Final.pdf [file ppat.1008529.s011.pdf]

Dear Dr. Parsek,

We thank you and the reviewers for your time reviewing our manuscript "Triclosan depletes the membrane potential in *Pseudomonas aeruginosa* biofilms inhibiting aminoglycoside induced adaptive resistance" in *PLOS Pathogens.* The reviewers made several excellent suggestions, which we incorporated to improve the manuscript. Specifically, we have made the following major changes:

1. We extensively edited and added additional information to the manuscript to address reviewer comments as can be seen by the track changes version of the manuscript that has been uploaded.
2. We added new data in Fig. S2 further demonstrating that we do not observe synergy between tobramycin and triclosan against exponentially growing planktonic cells.
3. We quantified CFUs in DPBS starved biofilms as show in Fig. S8 to demonstrate that these biofilms were starved for ATP.
4. We added an additional Fig. S4 to better clarify our flow cytometry gating strategy to quantify membrane potential.

Below, we specifically respond to each reviewer comment. We feel that our manuscript offers new insight into the role of protonophores and targeting membrane potential as new targets for antibiotic adjuvants to increase kill of biofilms. We hope you now find the manuscript suitable for publication in *PLOS Pathogens.* Please let me know if I can be of any further assistance.

Sincerely,
Chris Waters

Major Issues: Key Experiments Required for Acceptance

Reviewer #1:

Comment 1: In the initial finding by these authors, they show that triclosan is synergistic with tobramycin but only under biofilm conditions. There was no effect on planktonic cells. I would think that the effect of triclosan on RND efflux pumps is unlikely to be biofilm specific. Addition of why/how the triclosan effect is biofilm specific (in the model and the discussion) would improve the manuscript.

RESPONSE: As all reviewers expressed interest in the biofilm specific nature of the tobramycin-triclosan synergy, we extended our previous studies of this combination to assess its activity on planktonic cells. In our previous manuscript, we performed a MIC serial dilution experiment of tobramycin or tobramycin/triclosan against planktonic cells. Importantly, both agents were diluted at the same time. For this manuscript, we repeated the same basic experiment but maintained triclosan concentrations high at 100 μM in the presence of decreasing concentrations of tobramycin. The results confirm our prior finding that triclosan does not

enhance tobramycin killing of planktonic cells although it did decrease maximum culture yield in a tobramycin independent fashion. These data have now been included as Figure S2 in the manuscript.

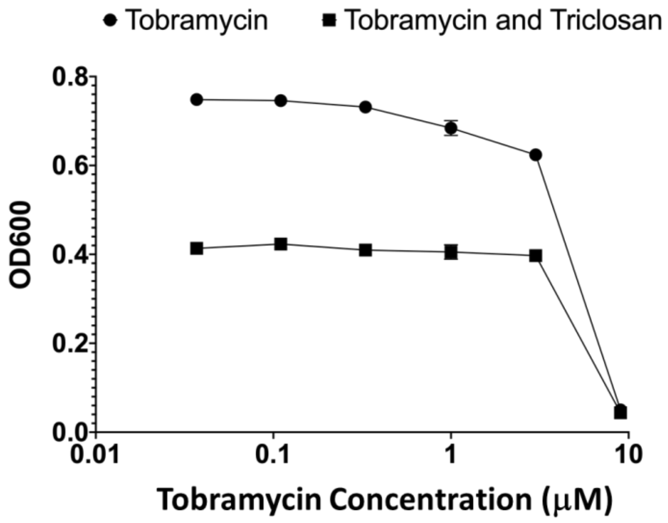

FIG S2. Triclosan does not enhance tobramycin killing of planktonic cells. Planktonic cells were treated with the indicated concentrations of tobramycin with or without 100 mM triclosan and grown for 16 hours before measuring the OD600. Error bars indicate the standard deviation.

To address the reviewer's comment, we now better explain our model for why we only see biofilm-specific activities. A key point is that adaptive resistance can be induced by the antibiotic concentration gradient generated in biofilms. In planktonic cells, there is not sufficient time for this induction and the cells are readily killed by tobramycin before triclosan can subvert this response. We now better develop these ideas in the discussion by the addition of the following text in lines 441-454:

> "Previously and reported here, we found triclosan does not synergize with tobramycin (or other aminoglycosides) against P. aeruginosa growing in exponential phase (Fig. S2)(30). Importantly, cells in exponential phase are highly susceptible to aminoglycosides and were eradicated by low μM of tobramycin, a finding in agreement with P. aeruginosa biofilms being ~1000-fold more tolerant to tobramycin than their planktonic counterparts (98). Alternatively, we found that the addition of triclosan enhanced tobramycin activity against cells in stationary phase (30), which is consistent with stationary cells being more phenotypically similar to biofilms (high cell density and low metabolic state) than cells in exponential phase (99). We hypothesize that because planktonic cells do not encounter the antimicrobial concentration gradients experienced by biofilm growing cells (100) and are rapidly killed without the required time for adaptive phenotypic changes to occur triclosan is unable to enhance tobramycin activity (4, 18). Furthermore, additional tolerance mechanisms found in cells growing as biofilms and cells in stationary phase that are not present in exponentially growing cells may be targeted by the combination."

COMMENT 2A: The interpretation of the whole-genome sequencing data is problematic. All 6 isolates that were sequenced from the two resistant populations fall into two classes. One class

(represented by F1_5, B2_30, and B2_82) contains substitutions leading to MexT P170L, FusA1 L40Q, PtsP A718P, and WspF Q132*. The other class (represented by F1_5, B2_8, and B2_57) contains a truncated MexT (80 bp deletion), FusA1 T64A, a truncated PtsP (1 bp deletion), WspF Q132*, PilY1 Q506*, and PmrB M292T. There are likely other genomic changes among the isolates within each classes that are the same. Therefore, although these isolates are not siblings, because the genetic changes are exactly the same within a class, they are definitely related. It suggests that the seeding population contained 2 isolates with these mutations at very low levels.

RESPONSE: The groups proposed by the reviewer are not quite correct, and to help the reader we have now included a table of the resistant mutants and their most relevant mutations embedded in Figure 2. All clones from population F1 (which was one well in a 96-well plate) developed a *fusA1* L40Q mutations while all clones from population B2 had a *fusA1* T64A mutation. Two different mutations were also observed in *ptsP* and *mexT*, but these were found in clones from both populations suggesting independent evolution. The only mutation common to all six clones was *wspF* Q132*. This raises the possibility that such a mutant was present in the founding population as a small subpopulation; however, given that we were only able to observe resistant populations in 2/9 of the gradual selection regimen, and 0/18 of the moderate or sudden selection regimen (meaning every population went extinct) argues that this mutation is insufficient to produce resistance to the combination. More likely, this mutation would be a hyper-biofilm former that was enriched during our selection regimen.

COMMENT 2B: The authors suggest that it is the FusA1 substitution (among all the changes) that is selected for in their passage experiment (lines 163-175, 382-390). However, it seems more likely that it is the WspF substitution that is selected for, since WspF mutations lead to cells that produce more robust biofilms (which, as mentioned by the authors, can increase the tolerance of biofilms against tobramycin) and the serial passage method used will likely select for hyperbiofilm formers. Furthermore, the FusA1 substitution is not the only change that would lead to increased tobramycin tolerance. While this is mentioned in the Discussion (lines 391-405), it is important to acknowledge that there is no way to know which change(s) were selected for. The strong focus on FusA1 (in the Results and the Discussion) is not warranted. Separately, if isolates of these two classes are at a low level in the seed population, it may be the reason why no resistant populations were found in the moderate regime.

RESPONSE: We agree with the reviewer that we focused too much on *fusA1* mutations in the original manuscript. We focused on *fusA1* because it is known that one of the *fusA1* mutations we isolated when present on the *P. aeruginosa* genome by itself promotes resistance to tobramycin (1), strongly suggesting that this mutation is causative for tobramycin resistance. But we agree with the reviewer that the other mutations could also be contributing to tobramycin resistance. Nevertheless, the bottom line is that the evolve clones exhibited increased resistance to tobramycin, increased sensitivity to triclosan, and resistance to the combination, showing that it is tobramycin driving killing in the combination. In response to the reviewer, we now more fully discuss the other mutations in the Results (lines 190-205), specifically stating, "Regardless of the mechanism, experimental evolution clearly demonstrates

these evolved isolates have more resistance to tobramycin but decreased resistance to the combination (Fig. 2), suggesting that killing by tobramycin is essential for the combination to be effective“. We removed this section from the discussion to reduce redundancy and shorten it.

COMMENT 3: In the ATP depletion experiment, it is not clear that the authors actually reduced the amount of ATP in the cells by starving the biofilms for 5 days. The stringent response should be activated by the shift to nutrient starvation, which would prevent a large drop in ATP levels. While there is less BacTiter-Glo signal (e.g. ATP concentration) in biofilms after starvation for 5 days (comparing the no treatment samples in Figs 2 and S6), this could be due to some level of dispersal of the biofilm so that there are fewer cells in the biofilm after 5 days of starvation (with no change in the ATP concentration per cell). To determine if ATP levels are different, the BacTiter-Glo signal should be normalized to the CFU for a starved and un-starved biofilm. Furthermore, this experiment should use CFUs (or some other measure of viability that does not rely on ATP concentrations) as a measure of total biofilm amount, so as to not confuse the variables in the experiment.

RESPONSE: We appreciate the concerns of the reviewer. The design for this experiment was inspired by a published study demonstrating that *P. aeruginosa* could survive for weeks suspended in water (2). Importantly, this study showed that *P. aeruginosa* did not show any significant death in water at one week (our experiment was performed in DPBS for five days). Moreover, they also used BacTiterGlo to show that ATP levels per cell decreased over time in nutrient starvation.

This previous study did not examine biofilms, and as suggested by the reviewer, it does remain possible that the biofilms could disperse during this 5-day incubation leading to less CFUs in the biofilm. To test this possibility, we quantified total CFUs of 24-hour old biofilms before and after incubation for 5 days in DPBS. The results, which are now included in the manuscript as Fig. S8, showed a 37% reduction in CFUs (a 1.5-fold decrease) in the DPBS starved biofilms although it was not statistically significant. Even if we consider this reduction to be a real difference, it cannot account for the 10-fold difference in BacTiterGlo that we observe in these biofilms. These data clearly indicate that the 5-day DPBS incubated biofilms had less ATP per cell and were in a starvation state. We respond to the suggestion of using CFUs to quantify biofilms rather than BacTiterGlo to Comment 4 by Reviewer 2.

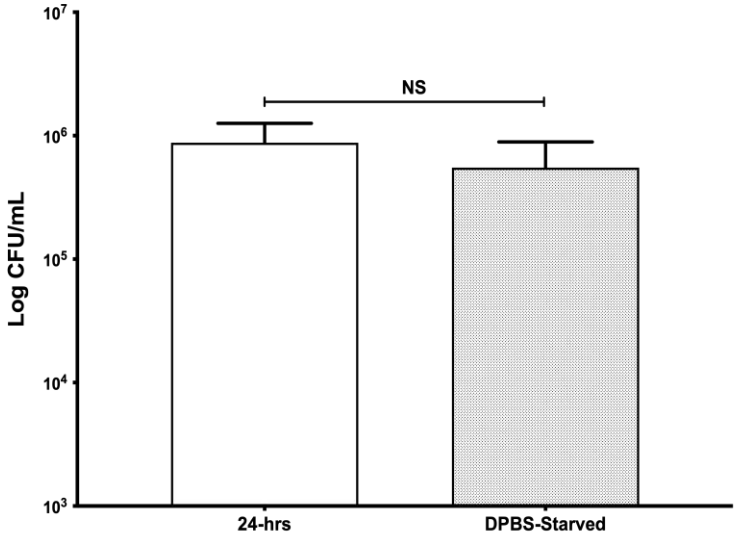

COMMENT 4: It is not clear why the authors chose Bonferroni's and Sidak's for their post-hoc statistical analyses. Bonferroni's (and Sidak's) is prone to Type II errors, which may explain why the authors see no statistical significance in some cases where samples look very different. For almost all the data, the samples are independent. Furthermore, means are being compared, so a Tukey HSD, which would reduce the Type II errors, could be used. In addition, instead of using asterisks to show statistical significance, letters to denote statistical groupings would be more informative. For instance, in Fig 2, it would allow for the within treatment comparison across strains.

RESPONSE: We agree with the reviewer and thank them for pointing this out. We are aware that Sidak's (more power than Bonferroni's) and Bonferroni's both can lead to type II errors. However, this statistical approach is valid because it is based on a priori knowledge in testing specific combinations. Importantly, the Bonferroni statistic is more rigorous than the Tukey's, which tolerates type I errors (3). We maintain that the statistical approach we used was more rigorous and better ensures that the conclusions about statistical significance we drew were correct. Nevertheless, the comments by the reviewer have motivated us to reconsider all our statistical analyses. We also considered using letter indicators to indicate statistically similar groups in Figure 2, but we felt that the key conclusion of those data is that the mutants are resistant to the combination. When we generated this figure with letter groupings as suggested, it is our opinion that comparison across groups is distracting from this key conclusion and offers no real scientific insight. Therefore, we prefer to leave the indication of statistical significance in Figure 2 as it was. Similarly, since the rest of the data contain few groups, we feel asterisks and bars were still appropriate and conveyed the significance clearly. The statistical analysis for each figure and rationale behind this approach is described below:

For Figure 1, we have re-run the analysis using Dunnett's multiple comparison, which is an appropriate approach given "no treatment" is a control group. That is, Dunnett's is a useful method to analyze studies having control groups (3). It is noted for being a powerful statistic capable of discovering small but significant differences among groups or combinations of groups (3). For Figure 1, we also ran Bonferroni's to compare specifically the difference between tobramycin vs the combination. And again, we found using Bonferroni's or Sidak's resulted in the same statistical outcome. At the reviewer's suggestion, we also performed Tukey's and again found the same statistical outcome.

For Figure 2, we do agree with the reviewer that Tukey's is applicable. We used Tukey's because we wanted to compare all groups to each other within each strain, having little a priori knowledge of how the strains will evolve and the various effects each treatment would have on them. Letters were also used in place of asterisks as requested.

For Figure 4, we again agreed with the reviewer and used Tukey's (panel A) and a t-test (panel B). Regardless of what analysis was used for these data, Donnette's, Sidak's or Bonferroni's the statistical results are the same.

For Figure 5, we again ran the statistics using Dunnett's, having a priori knowledge that "no treatment" is a control group and such analysis is appropriate (3). We then compared specifically using Bonferroni's tobramycin vs triclosan and tobramycin. Like Figure 1, we maintain that Dunnett's is the best test for this experimental design (3).

For Figure 6, we again chose Dunnett's because "no treatment" is our control group in this animal experiment and compared each treatment with this control group. We then specifically compared tobramycin vs the combination using Bonferroni's. We maintain that having the a priori that "no treatment" is a control group and our aim was to specifically test the difference between tobramycin vs the combination, such a statistical approach is appropriate (3).

Reviewer #2: There are no major issues with this manuscript.
RESPONSE: Thank you.

Reviewer #3: Many parts of this paper have merit. However, I have some concerns with the manuscript and some constructive criticism for the authors.

COMMENT 1: The first two points are related to integration of the findings with the literature. This manuscript is centered on aminoglycoside and triclosan synergy against biofilms, and the authors propose that bacterial energetics can be targeted to increase the susceptibility of P. aeruginosa biofilms to aminoglycosides (first stated in the abstract, Lines 35-37). So, do these bacterial energetics underlie the increased resistance of biofilms to antibiotics, or does the mechanism of synergy here apply to P. aeruginosa in planktonic cells in exponential or stationary phase too? The information and distinction are important. If this phenomenon does not occur for planktonic cells, then the authors' experiments might inform our basic understanding of biofilm microbiology. If these combinations are also effective against

planktonic cells, then I would suggest that some of the writing surrounding anti-biofilm activity should be constructed more carefully.

RESPONSE: Thank you for your question and we agree with the reviewer that our findings have implications for antibiotic tolerance mechanisms at different growth states. This question was also posed by Reviewer #1 in Comment 1, and we refer the reviewer to our response.

COMMENT 2: There is a lack of background information providing context to understand adaptive resistance in biofilms. There are still few reports describing how adaptive stress responses contribute to biofilm antimicrobial resistance phenotypes, and yet this information seems to be important for understanding the mechanism of antimicrobial synergy here. This information may also be important for distinguishing between planktonic and biofilm susceptibility phenotypes. Could the authors please add this information to help provide the reader with the salient information?

RESPONSE: As requested by the reviewer, more background information on adaptive resistance has been added to the introduction, and indeed it is the second paragraph of the manuscript in loines 61-78. We thank the reviewer for this comment as it has improved the focus of the manuscript.

> *"P. aeruginosa* also exhibits a unique and poorly understood form of inducible antibiotic resistance, known as "adaptive resistance", in response to cationic antimicrobials such as aminoglycosides, antimicrobial peptides, and polymyxins among others (6, 7). This form of resistance is transient and triggers an induction in gene expression, protein production, or alteration in antibiotic targets (7-22). Adaptive resistance results in alteration in the lipid A anchor of lipopolysaccharides (LPS) in response to polycationic antimicrobials due to the induction of regulatory proteins in classic two component systems like PhoP-PhoQ, PmrA-PmrB, CprR-CprS, and ParR-ParS (15-17). Another molecular mechanism leading to adaptive resistance is the induction of RND-type efflux pumps (11, 12, 18, 19). In several reports, RND-type efflux pumps are the major mediator of adaptive resistance, and they can be induced within 2-hrs of exposure to subinhibitory concentrations due to diffusion limitation in biofilms or sub-therapeutic dosing (11, 12, 18, 19, 23). Specifically, aminoglycosides cause the mistranslation of the PA5471 leader peptide PA5471.1, a peptide which inhibits the MexZ repressor, thereby triggering the induction of the RND-type efflux pump MexXY (24). Although adaptive resistance is not exclusive to the biofilm lifestyle, it is induced by exposure to subinhibitory concentrations of antibiotics, which can occur in biofilms due to reduced diffusion of cationic antimicrobials (7-22). The phenomenon of adaptive resistance has been observed in vitro, in animal models, and in CF patients (8, 14, 20-22).

COMMENT 3: The authors need to add more explanation and information to assist the reader with rationale and interpretation of the results. I would propose that it may be difficult for some readers, including perhaps some senior trainees, to understand how the results inform mode-of-action. This is a point pertaining to communication, not the science. For example (and

there are other places too): A) Lines 155-162. Perhaps the authors could elaborate how different findings from these experiments can inform their interpretations, setting the reader up to follow along more easily? B) Line 241. Could the authors please describe how a "dissociable proton" from triclosan disrupts the delta-Psi of cell membranes? C) Line 249. Could the authors please provide a description of how the DiOC2(3) dye functions as an indicator of delta-psi?

RESPONSE: Thank you for this feedback. We have significant rewritten many sections of the manuscript to increase clarity as described below.

A) Lines 161-165, to address this concern the following has been added to elaborate how using evolution and re-sequencing can lead to determining the target(s) of a drug or combination of compounds (23). In addition, the selection of resistant mutants and a description of major SNPs identified has been greatly expanded to aid in the explanation for how these genetic adaptions inform us of the mechanism of action. The following text has been added:

> "After eliminating inhibition of fatty acid synthesis and enhanced permeabilization as a possible explanation for the mechanism of action of triclosan (30), we turned to an unbiased experimental evolution approach to identify this mechanism (51). The principle behind this approach is that one can select for resistance to a given drug or combination and then perform re-sequencing to identify the intracellular genetic target(s)."

B) Line272-276: Dissociable protons are known to disrupt the delta-Psi of the cytoplasm of cells by increasing the concentration of protons inside the cell, where they are normally kept at a low concentration. We have now added a reference to a book chapter on how ionophores, protonophores or uncouplers disrupt the delta-Psi of cells. It is also important to point out the careful studies done by Teplova et al., who did detailed analyses of triclosan's ability to collapse the delta-Psi in a variety of organisms (25-29). The following text has been added:

> "Triclosan has a hydroxyl group with a dissociable proton that can disrupt the Δψ in eukaryotic cells, mitochondria, and bacteria (34-38). Protonophores have a dissociable proton and can pass through lipid bilayers as a conjugate base, shuttling protons across cellular membranes (63). This activity ultimately leads to a depletion in the Δψ by reducing the charge separation across the inner membrane (39, 40)."

C) Lines 282-288: The following has been added in regard to how DiOC2(3) dye functions as an indicator of delta-psi. This information is additionally detailed in the methods.

> "To measure changes in the Δψ of P. aeruginosa growing as biofilms, cells were stained with the fluorescent Δψ indicator DiOC2(3) dye and analyzed by flow cytometry. Cells were also co-stained with the membrane impermeant TO-PRO™-3 iodide dye to exclude permeabilized cells from analysis of Δψ. The DiOC2(3) dye emits in the fluorescein isothiocyanate (FITC) channel within all cells. However, greater membrane potential

drives accumulation and stacking of the dye in the cell cytoplasm, shifting emission to the phycoerythrin (PE) channel (see Fig. S4 for the flow cytometry gating strategy)."

COMMENT 4: A concern is drawing conclusions from results is the use of BacTiter-Glo. Caution is prudent. While it is established that viable cell counts correlate nicely with [ATP] for healthy cells, there are many potential reasons why this relationship might breakdown after a toxic antimicrobial exposure. It seems wise to repeat some of the key findings using viable cell counts to be sure of the results with BacTiter-Glo, especially since the mode of action of triclosan is thought to be via action on the delta-psi, which energizes ATP synthesis. In other words, there could be a decrease in intracellular [ATP] that does not correlate with an increase in log-killing. Also, since the authors are measuring [ATP], statements in the results about log-killing strike me as a leap in logic. As an alternative, one possible solution would be to make these statements in terms of observing changes in [ATP], rather than log-reductions in cell viability.

RESPONSE: We agree with the reviewer that caution is necessary, and we too had concerns and first began our work by establishing a calibration curve where CFUs and log luminescent values were plotted against each other and a linear regression performed (24). We found a strong correlation between CFUs and Bac-Titer Glo luminescent ($r2$ = 0.9884). We are confident that BacTiter-Glo is reflective of cell killing based on the following three lines of evidence. Given these various approaches providing data that validates the Bac-Titer Glo® data sets, we are confident in stating the data as Log killing.

1. First, CFUs were performed on cells in stationary phase in our initial publication (24), which are considered phenotypically similar to biofilms due to a combination of high cell density, increased release of quorum sensing molecules, and the rise of persister cells or those in a low metabolic state (33). We now describe these experiments in the discussion. These experiments support the Bac-Titer Glo® data, and are reprinted here for the reviewer's consideration:

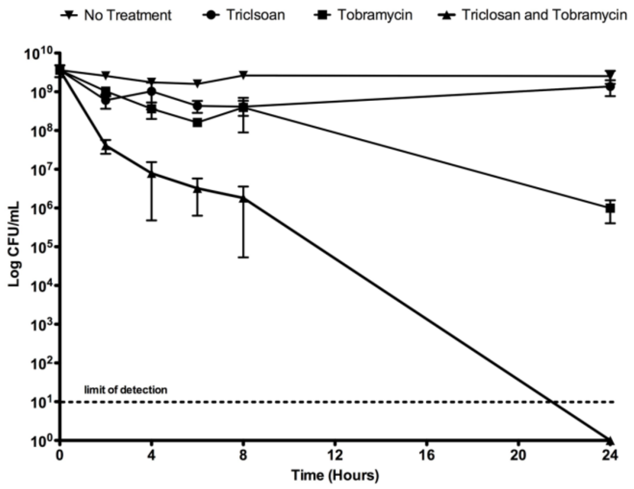

Tobramycin combined with triclosan kills persister cells. 20-hr old stationary-phase cells were treated with triclosan (100 µM) or tobramycin (500 µM) alone and in combination for 6-hrs. At 0, 2, 4, 6, 8, and 24-hrs aliquots were taken for CFUs/mL enumeration. The experiment was performed three times in triplicate. The results represent means plus the SEM.

2. In our initial publication (24), mature 24-hr biofilms grown in a flow cell were treated with tobramycin or triclosan alone and in combination and then stained with the standard live/dead stain (propidium iodide and SYTO9 green). Again, we found increase PI staining with the addition of triclosan to tobramycin, which was not observed with triclosan or tobramycin alone (24). This figure is shown here for the reviewer's consideration:

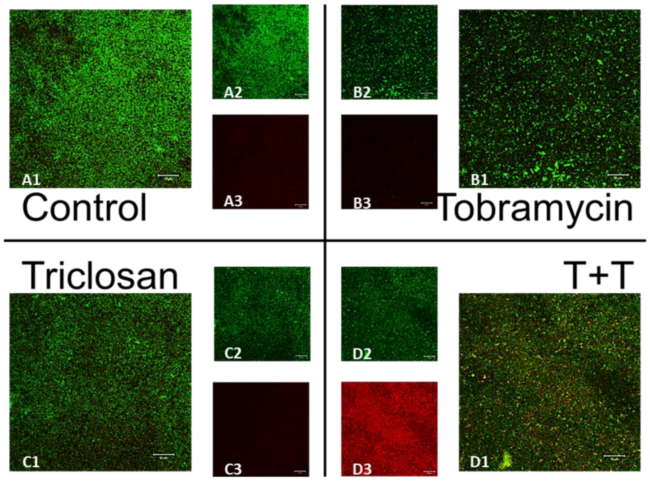

24-hr old biofilms grown in flow cells were treated with triclosan (100 µM), tobramycin (524 µM), or the combination for 6-hrs. Live cells are stained green and dead cells are stained red. Representative images are shown: no treatment (top left panel), tobramycin alone (top right panel), triclosan alone (lower left panel), combination (lower right panel). Inserts are shown for the live channel (A2, B2, C2, D2) and for the dead channel (A3, B3, C3, D3) for each condition.

3. Finally, new data in Fig. 1 of this manuscript describes flow cytometry experiments with the standard live/dead stain TO-PRO™-3 iodide which used to identify cells that had been permeabilized (Fig. 1). Importantly, this stain can only fluoresce in cells that have lost their cell membrane integrity and are therefore dead. Again, we recapitulated the BacTiter-Glo® data finding significant permeabilization when triclosan and tobramycin were used in combination at 2-hrs (Fig. 1).

COMMENT 5: It seems that some thought has been put into use of statistics. However, why do the authors plot standard error of the mean (SEM) as opposed to standard deviation (SD)? SEM values will always be smaller than SD values because of how SEM is calculated. However, SD is the statistical measure used to show variation among values within experiments. The use of SEM is unusual, especially for susceptibility testing. There is some long-standing humour among statisticians about how biologists use SEM (http://pmean.com/05/StandardError.html). I am concerned that SEM might be misleading and may not be the right choice here.

RESPONSE: Thank you, all figures have now been replaced with SD, as requested, and we describe our rationale for the statistical analysis approaches in response to Reviewer 1, Comment 4.

COMMENT 5: Could the authors also expand on the background information that RND-type efflux pumps are responsible for the multidrug resistance and tolerance of P. aeruginosa biofilms as opposed to planktonic cells? Evidence dating back nearly 20 y suggests that these pumps may not be the main culprits behind the reduced tolerance of P. aeruginosa biofilms to

antibiotics (ex. https://www.ncbi.nlm.nih.gov/pubmed/11353623). Additionally, a way of providing more supporting evidence for the mode of action proposed here would be to test strains with mutations in the RND-efflux pumps. Even if there is a hypersensitivity phenotype, antimicrobial synergy should be lost.

RESPONSE: Thank you for suggesting this addition. In the discussion we have expanded on the role of efflux pumps in biofilms and the contradictory publications surrounding their role in biofilm tolerance. In the Discussion, the following text has been added (lines 419-440:

> “The role of efflux pumps in antimicrobial resistance of *P. aeruginosa* biofilms has been debated. Initial studies from nearly 20 years ago found MexAB-OprM, MexCD-OprJ, MexEF-OprN and MexXY-OprM systems were not highly expressed in *P. aeruginosa* biofilms (85). However, these experiments showed heterogenous expression with cells closest to the substrate demonstrating the highest expression of these efflux pump systems (85), which is expected in biofilms due to biologically distinct subpopulations and the fact that the expression of MexCD-OprJ and MexEF-OprN are induced by their substrates (86). Contradicting these results, subsequent studies found efflux pumps are essential for both biofilm formation and tolerance. For example, MexAB-OprM and MexCD-OprJ are required for biofilm formation in the presences of macrolides (87) and MexCD-OprJ and MexAB-OprM are biofilm-specific defense mechanisms against azithromycin and colistin, respectively (88, 89). The role of RND-type efflux pumps in biofilm antibiotic tolerance was confirmed by a transposon mutant screen, which found the PA1874-1877 operon was responsible for biofilm-specific resistance to antibiotics and the expression of PA1874 is 10-fold higher in biofilms than planktonic cells (90). Further, Sauer and colleagues found a molecular link between efflux pump expression and the biofilm phenotype (91-93). They conducted several studies, finding that the BrlR transcription factor responds to changes in the concentrations of c-di-GMP (94, 95) and is required for the maximal expression of MexAB-OprM and MexEF-OprN efflux pumps in biofilms (91-93). However, work by Folsom and Stewart et al. found no evidence that efflux pumps promote biofilm antibiotic tolerance in *P. aeruginosa* (96, 97). It is likely these contradictory findings highlight the complex nature of phenotypic heterogeneity in biofilms, and biofilm physiology is highly dependent on experimental conditions. “

We agree that testing a strain with mutations in RND-efflux pumps will aid in supporting the mechanism of action. Work in the lab is currently underway to systematically knock-out each efflux pump, but *P. aeruginosa* is reported to have at least 12 RND-Type efflux pumps (47). However, as we expect multiple efflux pumps to contribute to the adaptive resistance we observe here, and furthermore disruption of one pump may impact the activity of others leading to different contributions to adaptive resistance given different genetic backgrounds, answering this question will require its own careful study, and we argue it is outside the scope of this manuscript whose major conclusion is disruption of the membrane potential in *P. aeruginosa* biofilms potentiates the bacteria to tobramycin killing.

Part III – Minor Issues: Editorial and Data Presentation Modifications

Reviewer #1:
COMMENT 1: The section on methyl-triclosan seems superfluous. Since the addition of the methyl group affects both activities of triclosan (dropping the membrane potential and inhibiting fatty acid synthesis), I do not see what this data adds to the manuscript. I also disagree that it supports the model (line 315), since it does not help differentiate between the different effects that triclosan is known to have.

RESPONSE: We respectfully disagree and maintain that this control is important, and we note that Reviewer #2 was enthusiastic about these studies. The experiments with methyl-triclosan are not intended to differentiate between FabI inhibition and protonophore activity. In this paper and our previous publication we provide multiple lines of evidence to demonstrate that inhibition of fatty acid synthesis does not lead to tobramycin synergy (24). Furthermore, because *P. aeruginosa* encodes for FabV (48), it is inherently resistant to the inhibition of fatty acid synthesis by triclosan, even if FabI is inhibited. Rather, testing of methyl-triclosan allowed us to use a chemical probe approach to determine if the protonophoric activity of triclosan was necessary for synergy, and our results clearly demonstrated it was.

COMMENT 2: There was no synergy when cells were treated with triclosan first (Fig 3), and the extrusion experiment shows approximately a 10-fold drop in both samples (with and without triclosan) during recovery (Fig 4). These data suggest that the triclosan-induced effect is quickly fixed by the cells as soon as the drug is removed from the extracellular environment. Why this would be the case is difficult to explain using the model presented. Why do the authors think that cells recover so quickly after triclosan removal?

RESPONSE: We actually find this result to be quite consistent with our data and model. First, in our initial study, performing a checkerboard dose response experiment demonstrated that relatively high levels of triclosan could enhance the activity of a low concentration of tobramycin, but the reverse was not true (see figure below). Therefore, we know we need high concentrations of triclosan for activity, and once triclosan is removed its concentrations likely drops below the limit of activity. Secondly, our model would predict that once triclosan is removed, the cells should be able to quickly reestablish a membrane potential to induce adaptive resistance once exposed to tobramycin. We thus would expect to see little killing in this condition, which is what is observed. What did surprise us was the high amount of killing observed when tobramycin was added first and removed before addition of triclosan. As described in the manuscript, it is known that tobramycin can associate with the biofilm matrix, and thus it seems that there is sufficient tobramycin to be enhanced by the high concentration of triclosan. Again, this is consistent with our checkerboard assay shown below. We speculate that the low levels of tobramycin that remain are sufficient, in the presence of triclosan, to corrupt membrane protein translation, initiating the EDP-II phase of tobramycin uptake.

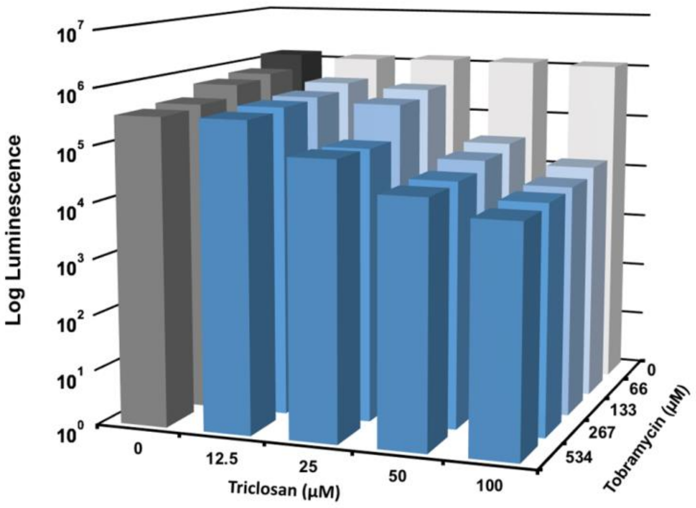

Triclosan enhances low concentrations of tobramycin. 24-hr old biofilms grown on MBEC plates were treated for 6-hrs with checkerboard dilutions of triclosan combined with tobramycin. Number of viable cells within the biofilms were quantified by BacTiter-GloTM. The assay was performed at least three times in triplicate. The results represent means.

COMMENT 3: It is not clear from the methods how the air-liquid interface biofilms are washed. Removing/exchanging the liquid underneath the biofilm would disturb the biofilm, no? Also in experiments that are not using the MBEC peg lids, how do the authors avoid the surface-liquid biofilm? In general, how is the air-liquid biofilm isolated?

RESPONSE: Thank you for your question. Air-liquid biofilms are formed as rings around a glass test tube or plastic 96-well plate, not as a pellicle. This allows for the removal or exchange of media without disruption of the biofilms. The image shown below using a hyper biofilm producer to illustrate the biofilm formed as a ring on a glass test tube (red arrow), where “spent” media has been removed to aid in visualization.

a. 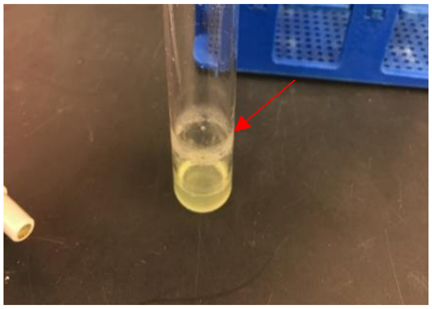

COMMENT 4: Inclusion of the flow cytometry graphs with the gates in the Supplemental would be useful for interpretation of the graphical data.

RESPONSE: These have been added to Fig. S4.

COMMENT 5: Minor points of confusion
- Line 192: The use of “alternatively” is confusing, since it suggests that the sentence is an alternative explanation for why cells die after tobramycin has been removed. Perhaps “in comparison” could be used instead.

- Line 252 - 256: “maintaining a membrane potential x-fold” is difficult to parse. While it is easy to see the fold change in membrane potential between the samples in the figure, the sentences are difficult to understand because of the use of “maintaining.”

RESPONSE: The text has been modified to address these comments.

Reviewer #2:

COMMENT 1: For Figure 1 and the normalization values, it is not clear what constitutes an “analyzed event”. This is the percentage of green fluorescent cells that also fluoresce red from FACS data? In the methods section, there is a lot of detail, but it is difficult to apply this to interpretation of the presented results.

RESPONSE: Yes, this is the percentage of cells excluding debris and those that stain negative for TO-PRO™-3 iodide. A representative gating strategy is now shown in Fig. S4, which hopefully makes interpreting this easier.

COMMENT 2: Line 152: “After 30 passages, two resistant populations from the gradual treatment regimen were isolated (Table 1)…” The two populations are F1 and B2? As one compares this paragraph with Table 1, it is difficult to reconcile them as they have no common notation between them.

RESPONSE: We realize this is confusing, the reason they are call F1 or B2 is because they came from these locations on a 96 well plate. We apologize for this, but the rationale for maintain this nomenclature is so in the future we can return to saved glycerol stocks of these 96-well plates.

a.
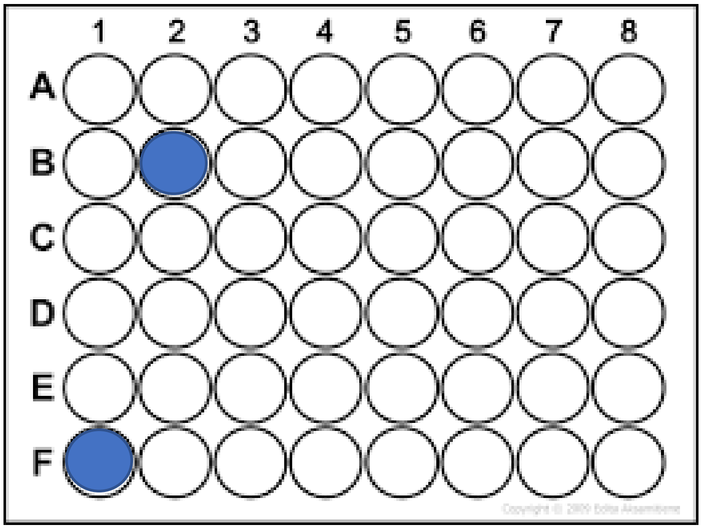

We have also modified the text to point out that we isolated individual resistant mutants from either the F_1 population or the B_2 population in line 180:

> "After 30 passages, two resistant populations from the gradual treatment regimen were isolated, F_1 and B_2 (Table 1), and 96-colonies were isolated from each mutant pool that were found to be resistant to the combination."

We have also added explanation to Table 1, detailing the naming scheme to help clarify confusion: Resistant mutant naming scheme: Cycle_Location in a 96 well plate_Individual mutant number

COMMENT 3: What is the significance of the yellow highlighting of Cycle 16 in Table S1? This appears to be a set of target concentrations that were tested, however, it is not the point at which either the gradual or moderate experiments stopped. Also, it is not clear how "gradual" and "moderate" are the best descriptors. Both terms could easily be applied to both sets if one considers either the initial concentration or the rate of change, yet moderate seems to describe initial starting concentrations while gradual describes a rate of change. Certainly "sudden" describes both accurately.

RESPONSE: The yellow highlighting indicates the minimum inhibitory concentration of tobramycin against planktonic cells. It is now removed from cycle 30, in which the experiment ended. Gradual and moderate are used to describe the fact that one treatment series approach the MIC more gradually than the other, in that it was exposed to fold dilutions of triclosan and tobramycin reaching the MIC after 16 cycles, whereas moderate reached the MIC after 8 cycles. We think these names are appropriate and argue to keep them.

COMMENT 4: Line 267. This section is great. An excellent control for the prior triclosan experiments.

RESPONSE: Thank you.

COMMENT 5: Line 279. It is not clear that the authors can specifically claim association with "ATP depletion" here. Certainly, an ability to generate ATP would be associated with these nutrient starved conditions, but it would be more reasonable to assign these 5-day DPBS experiments as "metabolically inactive" or some more general description, which would also include an ATP depleted state.

RESPONSE: The other two reviewers have raised concern over this experiment, and we have addressed it above. As we have shown that the CFUs remain unchanged after 5 days of incubation in DPBS but the BacTiterGlo signal is reduced 10-fold we argue the term "ATP depletion" is accurate.

COMMENT 6: Line 312. "occurring at 2-hrs" This descriptor is correct because it is when the authors performed their experiment. However, the statement is misleading in that it suggests the authors have delineated a timecourse for tricolosan action—which was not done here.

RESPONSE: We apologize for this confusion. We previously performed kinetic experiments and established a time course for synergy, finding triclosan accelerated killing to 2-hours from 6-hrs with tobramycin alone (24), shown below. The text has been modified to include a reference to our previous work in lines 358-360: "Triclosan accomplishes this by inhibiting a tobramycin induced surge in Δψ occurring at 2-hrs (Fig. 5), which leads to biofilm resistance to tobramycin as we have previously demonstrated (30)."

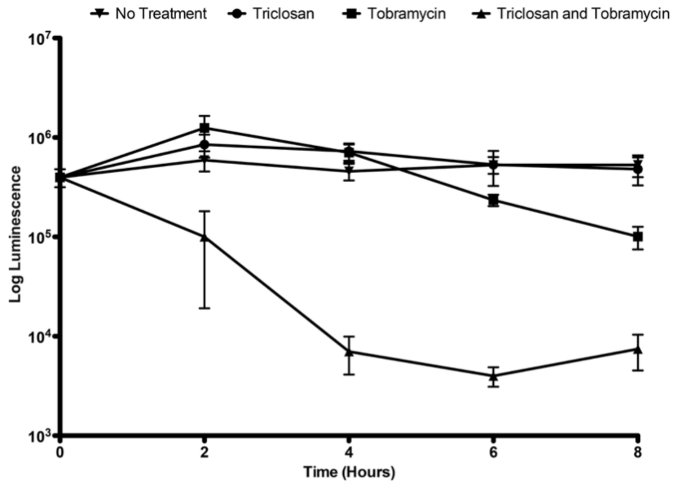

Triclosan enhances the onset and maximum efficacy of tobramycin. 24-hr old biofilms grown on MBEC plates were treated with triclosan (100 µM) or tobramycin (500 µM) alone and in combination. At 0, 2, 4, 6, and 8-hrs the number of viable cells within the biofilms were determined by BacTiter-GloTM. The assay was performed at least three times in triplicate. The results represent means plus the SEM.

Reviewer #3:

COMMENT 1: Line 86. Could the authors qualify the statement that the combination of triclosan and tobramycin is "100-times more effective." Do you mean that there is a 100-fold decrease in MBC, or that killing at particular concentrations is 100-times greater?

RESPONSE: We mean killing at that concentration was 100x greater (24), we observed a decrease from 1E6 to 1E4 in BacTiter-Glo signal.

COMMENT: Fig. 2. Could the authors put genotypes in the figure labels instead of strain names? This would be much more informative to the reader.

RESPONSE: Thank you for this excellent suggestion, they have been added to the figure.

COMMENT: The authors could consider depositing genome sequencing information in Genbank and providing Bioproject or Biosample IDs in the manuscript.

RESPONSE: These genome sequences have now been uploaded at the NCBI with BioSample accessions: SAMN15325472, SAMN15325473, SAMN15325474, SAMN15325475,

SAMN15325476, SAMN15325477, SAMN15325478, SAMN15325479, SAMN15325480, SAMN15325481.

References:

1. Bolard A, Plesiat P, Jeannot K. Mutations in Gene fusA1 as a Novel Mechanism of Aminoglycoside Resistance in Clinical Strains of Pseudomonas aeruginosa. Antimicrob Agents Chemother. 2018;62(2).
2. Lewenza S, Abboud J, Poon K, Kobryn M, Humplik I, Bell JR, et al. Pseudomonas aeruginosa displays a dormancy phenotype during long-term survival in water. PLoS One. 2018;13(9):e0198384.
3. Lee S, Lee DK. What is the proper way to apply the multiple comparison test? Korean J Anesthesiol. 2018;71(5):353-60.
4. Yamamoto M, Ueda A, Kudo M, Matsuo Y, Fukushima J, Nakae T, et al. Role of MexZ and PA5471 in transcriptional regulation of mexXY in Pseudomonas aeruginosa. Microbiology. 2009;155(Pt 10):3312-21.
5. Morita Y, Tomida J, Kawamura Y. Responses of Pseudomonas aeruginosa to antimicrobials. Front Microbiol. 2014;4.
6. Barclay ML, Begg EJ, Chambers ST, Thornley PE, Pattemore PK, Grimwood K. Adaptive resistance to tobramycin in Pseudomonas aeruginosa lung infection in cystic fibrosis. J Antimicrob Chemother. 1996;37(6):1155-64.
7. Barclay ML, Begg EJ, Chambers ST. Adaptive resistance following single doses of gentamicin in a dynamic in vitro model. Antimicrob Agents Chemother. 1992;36(9):1951-7.
8. Fernández L, Breidenstein EBM, Hancock REW. Creeping baselines and adaptive resistance to antibiotics. Drug Resistance Updates. 2011;14(1):1-21.
9. Fernández L, Gooderham WJ, Bains M, McPhee JB, Wiegand I, Hancock RE. Adaptive resistance to the "last hope" antibiotics polymyxin B and colistin in Pseudomonas aeruginosa is mediated by the novel two-component regulatory system ParR-ParS. Antimicrob Agents Chemother. 2010;54(8):3372-82.
10. Jeannot K, Sobel ML, El Garch F, Poole K, Plesiat P. Induction of the MexXY efflux pump in Pseudomonas aeruginosa is dependent on drug-ribosome interaction. J Bacteriol. 2005;187(15):5341-6.
11. Karlowsky JA, Saunders MH, Harding GA, Hoban DJ, Zhanel GG. In vitro characterization of aminoglycoside adaptive resistance in Pseudomonas aeruginosa. Antimicrob Agents Chemother. 1996;40(6):1387-93.
12. Pagedar A, Singh J, Batish VK. Efflux mediated adaptive and cross resistance to ciprofloxacin and benzalkonium chloride in Pseudomonas aeruginosa of dairy origin. J Basic Microbiol. 2011;51(3):289-95.
13. Xiong YQ, Caillon J, Kergueris MF, Drugeon H, Baron D, Potel G, et al. Adaptive resistance of Pseudomonas aeruginosa induced by aminoglycosides and killing kinetics in a rabbit endocarditis model. Antimicrobial agents and chemotherapy. 1997;41(4):823-6.
14. Barrow K, Kwon DH. Alterations in two-component regulatory systems of phoPQ and pmrAB are associated with polymyxin B resistance in clinical isolates of Pseudomonas aeruginosa. Antimicrob Agents Chemother. 2009;53(12):5150-4.

15. Fernández L, Jenssen H, Bains M, Wiegand I, Gooderham WJ, Hancock RE. The two-component system CprRS senses cationic peptides and triggers adaptive resistance in Pseudomonas aeruginosa independently of ParRS. Antimicrob Agents Chemother. 2012;56(12):6212-22.
16. Moore RA, Bates NC, Hancock RE. Interaction of polycationic antibiotics with Pseudomonas aeruginosa lipopolysaccharide and lipid A studied by using dansyl-polymyxin. Antimicrobial agents and chemotherapy. 1986;29(3):496-500.
17. Hay T, Fraud S, Lau CH, Gilmour C, Poole K. Antibiotic inducibility of the mexXY multidrug efflux operon of Pseudomonas aeruginosa: involvement of the MexZ anti-repressor ArmZ. PLoS One. 2013;8(2):e56858.
18. Hocquet D, Vogne C, El Garch F, Vejux A, Gotoh N, Lee A, et al. MexXY-OprM efflux pump is necessary for a adaptive resistance of Pseudomonas aeruginosa to aminoglycosides. Antimicrobial agents and chemotherapy. 2003;47(4):1371-5.
19. Barclay ML, Begg EJ. Aminoglycoside adaptive resistance: importance for effective dosage regimens. Drugs. 2001;61(6):713-21.
20. Barclay ML, Begg EJ, Chambers ST, Peddie BA. The effect of aminoglycoside-induced adaptive resistance on the antibacterial activity of other antibiotics against Pseudomonas aeruginosa in vitro. J Antimicrob Chemother. 1996;38(5):853-8.
21. Daikos GL, Lolans VT, Jackson GG. First-exposure adaptive resistance to aminoglycoside antibiotics in vivo with meaning for optimal clinical use. Antimicrob Agents Chemother. 1991;35(1):117-23.
22. Sandoval-Motta S, Aldana M. Adaptive resistance to antibiotics in bacteria: a systems biology perspective. Wiley Interdiscip Rev Syst Biol Med. 2016;8(3):253-67.
23. Kofler R, Schlotterer C. A guide for the design of evolve and resequencing studies. Mol Biol Evol. 2014;31(2):474-83.
24. Maiden MM, Hunt AMA, Zachos MP, Gibson JA, Hurwitz ME, Mulks MH, et al. Triclosan Is an Aminoglycoside Adjuvant for Eradication of Pseudomonas aeruginosa Biofilms. Antimicrob Agents Chemother. 2018;62(6):e00146-18.
25. Yoon DS, Choi Y, Cha DS, Zhang P, Choi SM, Alfhili MA, et al. Triclosan Disrupts SKN-1/Nrf2-Mediated Oxidative Stress Response in C. elegans and Human Mesenchymal Stem Cells. Sci Rep. 2017;7(1):12592.
26. Weatherly LM, Shim J, Hashmi HN, Kennedy RH, Hess ST, Gosse JA. Antimicrobial agent triclosan is a proton ionophore uncoupler of mitochondria in living rat and human mast cells and in primary human keratinocytes. J Appl Toxicol. 2016;36(6):777-89.
27. Popova LB, Nosikova ES, Kotova EA, Tarasova EO, Nazarov PA, Khailova LS, et al. Protonophoric action of triclosan causes calcium efflux from mitochondria, plasma membrane depolarization and bursts of miniature end-plate potentials. Biochim Biophys Acta Biomembr. 2018;1860(5):1000-7.
28. Teplova VV, Belosludtsev KN, Kruglov AG. Mechanism of triclosan toxicity: Mitochondrial dysfunction including complex II inhibition, superoxide release and uncoupling of oxidative phosphorylation. Toxicol Lett. 2017;275:108-17.
29. Ajao C, Andersson MA, Teplova VV, Nagy S, Gahmberg CG, Andersson LC, et al. Mitochondrial toxicity of triclosan on mammalian cells. Toxicol Rep. 2015;2:624-37.

30. Nicholls DG, Ferguson SJ. Ion Transport Across Energy-Conserving Membranes. In: Nicholls DG, Ferguson SJ, editors. Bioenergetics. Boston: Academic Press; 2013. p. 13-25.
31. Mitchell P. Coupling of phosphorylation to electron and hydrogen transfer by a chemi-osmotic type of mechanism. Nature. 1961;191(4784):144-8.
32. Mitchell P. Chemiosmotic coupling in oxidative and photosynthetic phosphorylation. 1966. Biochim Biophys Acta. 2011;1807(12):1507-38.
33. Spoering AL, Lewis K. Biofilms and planktonic cells of Pseudomonas aeruginosa have similar resistance to killing by antimicrobials. Journal of bacteriology. 2001;183(23):6746-51.
34. De Kievit TR, Parkins MD, Gillis RJ, Srikumar R, Ceri H, Poole K, et al. Multidrug efflux pumps: expression patterns and contribution to antibiotic resistance in Pseudomonas aeruginosa biofilms. Antimicrob Agents Chemother. 2001;45(6):1761-70.
35. Koo SH. Overexpression of Efflux Pump in Multiresistant Pseudomonas aeruginosa: How You Will Discover and Treat It? Infect Chemother. 2015;47(2):142-4.
36. Alav I, Sutton JM, Rahman KM. Role of bacterial efflux pumps in biofilm formation. J Antimicrob Chemother. 2018;73(8):2003-20.
37. Gillis RJ, White KG, Choi KH, Wagner VE, Schweizer HP, Iglewski BH. Molecular basis of azithromycin-resistant Pseudomonas aeruginosa biofilms. Antimicrob Agents Chemother. 2005;49(9):3858-67.
38. Pamp SJ, Gjermansen M, Johansen HK, Tolker-Nielsen T. Tolerance to the antimicrobial peptide colistin in Pseudomonas aeruginosa biofilms is linked to metabolically active cells, and depends on the pmr and mexAB-oprM genes. Mol Microbiol. 2008;68(1):223-40.
39. Zhang L, Mah TF. Involvement of a novel efflux system in biofilm-specific resistance to antibiotics. J Bacteriol. 2008;190(13):4447-52.
40. Liao J, Sauer K. The MerR-like transcriptional regulator BrlR contributes to Pseudomonas aeruginosa biofilm tolerance. J Bacteriol. 2012;194(18):4823-36.
41. Liao J, Schurr MJ, Sauer K. The MerR-like regulator BrlR confers biofilm tolerance by activating multidrug efflux pumps in Pseudomonas aeruginosa biofilms. J Bacteriol. 2013;195(15):3352-63.
42. Chambers JR, Liao J, Schurr MJ, Sauer K. BrlR from Pseudomonas aeruginosa is a c-di-GMP-responsive transcription factor. Mol Microbiol. 2014;92(3):471-87.
43. Romling U, Galperin MY, Gomelsky M. Cyclic di-GMP: the first 25 years of a universal bacterial second messenger. Microbiol Mol Biol Rev. 2013;77(1):1-52.
44. Valentini M, Filloux A. Biofilms and Cyclic di-GMP (c-di-GMP) Signaling: Lessons from Pseudomonas aeruginosa and Other Bacteria. J Biol Chem. 2016;291(24):12547-55.
45. Folsom JP, Richards L, Pitts B, Roe F, Ehrlich GD, Parker A, et al. Physiology of Pseudomonas aeruginosa in biofilms as revealed by transcriptome analysis. BMC Microbiol. 2010;10:294.
46. Stewart PS, Franklin MJ, Williamson KS, Folsom JP, Boegli L, James GA. Contribution of stress responses to antibiotic tolerance in Pseudomonas aeruginosa biofilms. Antimicrob Agents Chemother. 2015;59(7):3838-47.
47. Poole K. Pseudomonas aeruginosa: resistance to the max. Front Microbiol. 2011;2:65.
48. Zhu L, Lin J, Ma J, Cronan JE, Wang H. Triclosan resistance of Pseudomonas aeruginosa PAO1 is due to FabV, a triclosan-resistant enoyl-acyl carrier protein reductase. Antimicrob Agents Chemother. 2010;54(2):689-98.
